# Infection-Triggered Immune Dysregulation and Immunopathology in Lyme Disease: Mechanisms and Clinical Implications

**DOI:** 10.3390/jcm15082922

**Published:** 2026-04-11

**Authors:** Klavio Pine, Vivian Pine, Nicoleta Negrut, Anca Ferician, Paula Marian

**Affiliations:** 1 Doctoral School of Biomedical Sciences, Faculty of Medicine and Pharmacy, University of Oradea, 410087 Oradea, Romania; pine.klavio@student.uoradea.ro; 2 Asclepion Clinic, 6 Cuejdi Street, 610024 Piatra Neamț, Romania; pine.vivian@yahoo.com; 3 Department of Psycho-Neuroscience and Recovery, Faculty of Medicine and Pharmacy, University of Oradea, 410073 Oradea, Romania; 4 Department of Medical Disciplines, Faculty of Medicine and Pharmacy, University of Oradea, 410073 Oradea, Romania; paulamarian@uoradea.ro

**Keywords:** Lyme disease, Borrelia burgdorferi, infection-triggered autoimmunity, type I interferon, Th17, molecular mimicry, peptidoglycan, immune-mediated inflammation, post-infectious syndromes

## Abstract

Lyme disease (LD) is classically defined as a tick-borne infection caused by *Borrelia burgdorferi sensu lato* (Bbsl). However, accumulating evidence indicates that, beyond microbial persistence, Bbsl infection can initiate sustained immune dysregulation and post-infectious inflammatory phenotypes in a subset of patients. This narrative review integrates open-access experimental, translational, and clinical data and discusses LD within the spectrum of infection-triggered, immune-mediated processes. We review key immunopathogenic mechanisms, including dysregulated innate immune activation, type I interferon (IFN-I) signaling, T helper 1 and T helper 17 (Th1/Th17) polarization with regulatory T-cell (Treg) insufficiency, antigen persistence (notably borrelial peptidoglycan), and pathways linking infection to autoimmunity such as molecular mimicry, epitope spreading, and human leukocyte antigen (HLA)-restricted susceptibility. These mechanisms are integrated with immune-mediated clinical manifestations affecting the central nervous system (CNS), peripheral nervous system (PNS), musculoskeletal system, heart, skin, and hematologic compartment. Finally, we discuss translational implications for diagnosis, biomarker-guided stratification, and emerging therapeutic strategies that extend beyond antimicrobial therapy, while addressing current controversies and limitations. This framework supports a mechanistic model in which Lyme disease-associated morbidity in selected patients reflects persistent immune activation and dysregulated host responses triggered by infection.

## 1. Introduction

Lyme disease (LD) is a multisystem condition caused by infection with *Borrelia burgdorferi sensu lato* (Bbsl) and transmitted by Ixodes ticks. Conventional pathogenic models primarily focus on microbial transmission, dissemination, immune evasion strategies, and eradication of the pathogen through antibiotic therapy as the principal determinants of disease outcome. While this paradigm adequately explains early manifestations of infection, it fails to account for the pronounced heterogeneity of clinical courses fully, the wide variability in tissue injury among similarly exposed individuals, and the persistence of inflammatory symptoms in a subset of patients despite guideline-concordant antimicrobial treatment [1,2,3]. In addition, early systemic involvement affecting multiple organs, including transient hepatic abnormalities reported in the initial Connecticut case series, further supports the concept that host immune responses contribute significantly to disease expression beyond localized infection, although the relative contribution of direct infection versus immune-mediated hepatocellular injury remains incompletely defined [4,5]. Post-treatment LD syndrome (PTLDS) refers to persistent symptoms such as fatigue, musculoskeletal pain, and cognitive impairment that continue for at least six months after appropriate antibiotic therapy in patients with documented LD and without evidence of active infection [6]. This entity should be distinguished from the term “chronic LD,” which is inconsistently defined and often used to imply ongoing infection despite a lack of supporting evidence.

Multiple interacting factors, including pathogen variability, coinfections, prior Bbsl exposure, host immune heterogeneity, and comorbidities, likely influence disease heterogeneity in LD.

Accumulating experimental and clinical data indicate that tissue damage in LD is driven predominantly by host immune responses, with limited evidence supporting direct cytotoxic effects of the spirochete itself. In many disease stages, spirochetal burden is low relative to the degree of inflammation and organ dysfunction, a discrepancy that is particularly evident in late manifestations such as Lyme arthritis (LA) and Lyme neuroborreliosis (LNB) [1,2,7]. In antibiotic-refractory or post-infectious LA, synovial hyperplasia, inflammatory infiltrates, and tissue remodeling may persist for prolonged periods despite microbiological clearance, supporting a model of impaired immune resolution instead of ongoing infection [7,8,9]. Persistent inflammation in this context may also reflect stable changes in synovial and stromal cell phenotypes that maintain inflammatory activity following the initial infectious trigger. On this basis, the present review adopts an immunopathogenic framework in which Borrelia infection serves as an initiating event that triggers sustained immune dysregulation in susceptible hosts. Emphasis is placed on altered innate and adaptive immune signaling, persistent antigenic stimulation, and infection-triggered autoimmune mechanisms that together promote chronic inflammation and post-infectious disease phenotypes. This perspective provides a biologically coherent explanation for symptom persistence and offers a rational foundation for diagnostic stratification and therapeutic strategies that extend beyond antimicrobial escalation alone.

This review aims to examine the immunopathogenic mechanisms underlying infection-triggered autoimmunity in Lyme disease and to discuss their clinical implications for diagnosis, disease stratification, and therapeutic strategies.

## 2. Materials and Methods

This work is a narrative, non-systematic review of the literature addressing the immunopathogenesis, clinical manifestations, and therapeutic implications of Bbsl infection. The systematic review methodology and PRISMA framework were not applied because the primary objective was a mechanistic synthesis instead of quantitative effect estimation. Relevant publications were identified through searches of PubMed/MEDLINE, Scopus, Web of Science, and the Cochrane Library, covering the period from January 2000 to December 2025. Search terms included combinations of “Lyme disease”, “Borrelia burgdorferi”, “immunopathogenesis”, “type I interferon”, “Th17”, “autoimmunity”, “antigen persistence”, “neuroborreliosis”, “Lyme arthritis”, and “post-treatment Lyme disease syndrome”, as well as related immunologic concepts such as “molecular mimicry”, “epitope spreading”, “bystander activation”, “idiotypic network”, and “innate immunity”. Additional sources were identified through manual screening of reference lists from key reviews and primary research articles. Peer-reviewed original studies, experimental models, clinical cohorts, and high-quality narrative or systematic reviews published in English were included based on relevance to immune mechanisms, methodological rigor, and translational insight. Articles lacking full-text availability, mechanistic relevance, or adequate clinical characterization were excluded.

## 3. Immunopathogenic Mechanisms, Clinical Phenotypes, and Therapeutic Implications in LD

### 3.1. Core Immunopathogenic Mechanisms Triggered by *Bbsl*

#### 3.1.1. Dysregulated Innate Immune Activation

Borrelial lipoproteins activate innate immunity primarily through Toll-like receptor 2 (TLR2) signaling, inducing sustained production of pro-inflammatory cytokines and chemokines [1]. Disease severity and chronicity correlate more strongly with the magnitude and persistence of innate immune activation than with spirochetal burden alone [1,2]. In a subset of patients, systems immunology analyses demonstrate prolonged inflammatory transcriptional signatures despite appropriate antibiotic therapy, indicating impaired resolution of innate immune responses with no evidence of ongoing bacterial replication [10]. Additionally, Bbsl exhibits morphological plasticity, including non-spiral forms and aggregate structures, which may facilitate persistence of immunogenic bacterial components and contribute to sustained innate immune activation. Persistent innate immune activation establishes a pro-inflammatory tissue environment that facilitates dysregulated adaptive immune responses and promotes post-infectious immunopathology [2,10].

#### 3.1.2. The Type I Interferon (IFN) Axis as an Inflammatory Amplifier

Type IFN-1 signaling represents a central immunopathological axis in LD, most prominently demonstrated in LA [3,6,8]. Experimental studies consistently show that genetic or pharmacologic disruption of IFN-I signaling markedly attenuates inflammatory tissue damage while exerting minimal effects on spirochetal burden, indicating that IFN-I activity directly mediates immune-driven pathology [11,12].

Mechanistically, Bbs*l* induces IFN-I responses through multiple innate sensing pathways, including recognition of bacterial ribonucleic acid (RNA) and activation of intracellular nucleic acid sensors such as the cyclic GMP–AMP synthase–stimulator of IFN genes (cGAS–STING) signaling axis [12,13,14,15]. These pathways initiate a transcriptional program that amplifies inflammatory signaling independently of bacterial load. Importantly, IFN-I-mediated inflammation extends beyond hematopoietic immune cells, as stromal populations—particularly fibroblasts and endothelial cells—contribute to sustained inflammation by producing IFN-inducible chemokines that promote ongoing leukocyte recruitment and tissue-level immune activation [12].

Human transcriptomic studies further support the pathogenic relevance of this axis, demonstrating IFN-stimulated gene expression profiles that correlate with inflammatory disease activity and typically decline in parallel with clinical improvement [15,16]. Collectively, these findings identify IFN-I signaling as a critical amplifier of inflammation in Lyme disease and provide a mechanistic framework explaining the persistence of tissue injury and clinical severity in certain phenotypes despite effective microbial clearance.

#### 3.1.3. Adaptive Immune Polarization: Pathogenic T Helper (Th) Th1/Th17 Responses and Regulatory T-Cell Failure

Adaptive immune responses in Lyme disease are characterized by a pronounced polarization toward inflammatory Th1 and Th17 T-cell programs within affected tissues, including the synovium and neural interfaces. Th17 responses are of particular immunopathological relevance, as they bridge antimicrobial effector functions with pathways that promote chronic inflammation and autoimmunity. Human studies demonstrate that Th17-associated cytokine signatures not only correlate with early anti-*Borrelia* immune responses but also associate with subsequent autoantibody development in patients who progress to post-infectious LA [17].

Sustained inflammation further reflects impaired regulatory control not merely exaggerated effector responses alone. Regulatory T cells (Tregs) play a critical role in terminating T-cell-mediated inflammation following pathogen clearance. Experimental models show that depletion or functional disruption of Tregs permits unchecked expansion of Th1 and Th17 populations, leading to persistent inflammatory disease marked by increased IFN-γ and interleukin-17 (IL-17) production despite effective microbial elimination [18].

Persistent recruitment, activation, and local retention of Th1 and Th17 cells are reinforced by inflammatory signaling established during early infection, stabilizing a T-cell-dominated pro-inflammatory tissue environment. These self-sustaining T-cell circuits closely parallel mechanisms described in autoimmune and chronic inflammatory disorders, providing a unifying immunopathological framework for prolonged T-cell activation in selected LD phenotypes [19,20].

#### 3.1.4. Antigen Persistence Without Viable Infection

A key element of immune-mediated disease in LD is the persistence of bacterial antigens despite successful eradication of viable organisms. Among these antigens, Bbs*l* peptidoglycan has emerged as a particularly important driver of sustained inflammation. Seminal studies demonstrated that peptidoglycan fragments can remain within the synovial tissue of patients with LA for prolonged periods following antibiotic therapy, where they function as a stable and biologically active inflammatory stimulus [21]. Unlike intact spirochetes, these residual bacterial components are resistant to antimicrobial clearance yet retain the capacity to engage host immune pathways. Subsequent investigations have shown that persistent peptidoglycan is sufficient to provoke both local and systemic immune activation, inducing cytokine responses and inflammatory programs that mirror those observed in chronic inflammatory disorders. Coinfections with other tick-borne pathogens have also been proposed as potential contributors to persistent inflammatory symptoms in some patients; however, their role remains uncertain and is subject to ongoing debate [22].

#### 3.1.5. Infection-Triggered Autoimmunity: Mechanistic Framework

Infectious agents are well-established triggers of autoimmune and immune-mediated disease, acting through mechanisms such as molecular mimicry, bystander immune activation, and epitope spreading, particularly when inflammation is intense or prolonged [23,24]. LD represents a biologically credible context for these processes, given the combination of sustained antigen exposure, strong cytokine-driven immune activation, and host genetic susceptibility. Genetic association studies in humans demonstrate that antibiotic-refractory LA is linked to specific HLA-DR alleles capable of efficiently presenting immunodominant *Borrelia* peptides, underscoring the role of antigen presentation in shaping post-infectious inflammatory outcomes [25]. Experimental and translational studies examining molecular mimicry between *Borrelia* outer surface proteins and host antigens further suggest that cross-reactive T-cell responses can arise [25,26,27]. However, the magnitude and clinical relevance of such cross-reactivity appear to vary substantially between individuals, likely reflecting differences in genetic background and immune regulation [25,26,27]. As inflammation persists, immune responses may broaden beyond the initial microbial targets through epitope spreading, leading to progressive diversification toward host antigens. Recent immunologic profiling studies in LA provide support for this process, demonstrating expansion of autoreactive immune responses over time [28,29]. Collectively, these observations support a model of infection-triggered autoimmunity without implying that LD constitutes a primary autoimmune disorder. Instead, *Borrelia* infection functions as an initiating event that, in susceptible hosts, precipitates maladaptive immune responses capable of sustaining pathology well beyond pathogen eradication.

The major immunopathogenic mechanisms contributing to post-infectious manifestations of Lyme disease are summarized in Table 1.

**Figure 1:**
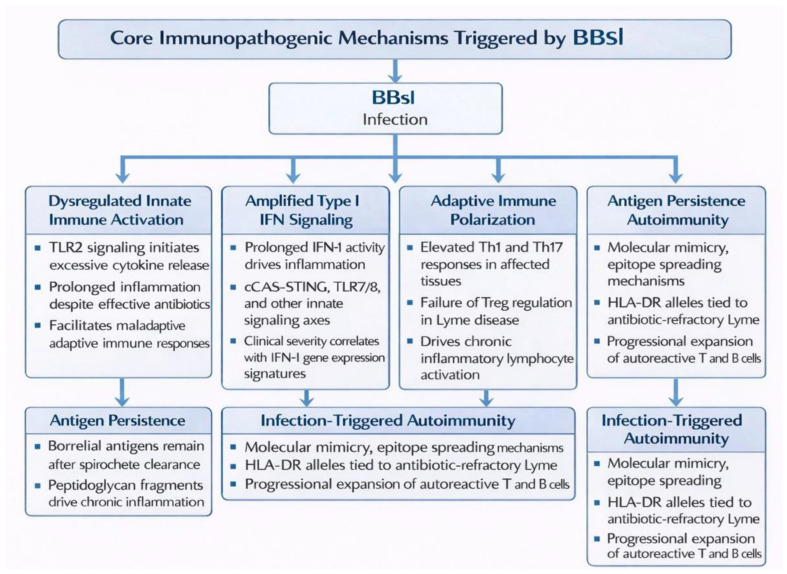
Core Immunopathogenic Mechanisms Triggered by *Bbsl*. *Bbsl*—*Borrelia burgdorferi sensu lato*; TLR—Toll-like receptor; IFN—interferon; Th1—T helper 1; Th17—T helper 17; Treg—regulatory T cell; HLA—human leukocyte antigen; cGAS–STING—cyclic GMP–AMP synthase–stimulator of interferon genes.

The conceptual model integrating the principal immunopathogenic mechanisms triggered by *Bbsl* is presented in Figure 1.

### 3.2. Immune-Mediated Clinical Phenotypes

#### 3.2.1. General Immunopathologic Framework of Nervous System Involvement

##### Central Nervous System (CNS) Involvement

LNB is characterized by CNS invasion by Bbsl followed by a pronounced intrathecal immune response. Spirochetal lipoproteins activate innate immune pathways via TLR2 signaling, inducing production of pro-inflammatory cytokines and chemokines, notably C-X-C motif chemokine ligand 13 (CXCL13), which drives massive B-cell recruitment into the cerebrospinal fluid (CSF). This results in lymphocytic pleocytosis, blood–brain barrier (BBB) dysfunction, and intrathecal synthesis of *Borrelia*-specific antibodies. Clinically, cranial neuropathies—particularly facial nerve palsy—represent one of the most common neurological manifestations of Lyme neuroborreliosis. Facial nerve involvement is thought to result from inflammatory processes affecting the nerve within the context of meningeal and radicular inflammation, reflecting both infection-associated and immune-mediated mechanisms [30]. While these mechanisms are essential for pathogen control, sustained immune activation may promote bystander neuronal injury, epitope spreading, and immune dysregulation, providing a biologically plausible link between infection and immune-mediated or autoimmune CNS manifestations reported in association with LD. Importantly, these processes do not imply persistent infection; they reflect post-infectious or immune-amplified neuroinflammation [30,31,32,33].

Within this immunopathologic framework, multiple sclerosis (MS)-like syndromes associated with LD describe clinical and radiologic presentations resembling MS, including focal neurological deficits, sensory disturbances, limb weakness, gait instability, fatigue, and cognitive impairment, accompanied by white-matter lesions on magnetic resonance imaging (MRI). These cases represent a significant diagnostic challenge, as nonspecific MRI abnormalities and incidental *Borrelia* seropositivity may lead to misdiagnosis. Evidence indicates that true demyelinating disease caused directly by Bbsl is rare, and LNB may mimic MS through inflammatory mechanisms or coexist coincidentally with primary demyelinating disorders [30,33].

From an immunopathologic perspective, MS-like manifestations in LNB are thought to arise from secondary immune-mediated injury, not primary demyelination. Persistent intrathecal inflammation, microglial activation, cytokine release, and BBB disruption may facilitate immune cell infiltration into white matter, producing reversible demyelination-like changes. Additionally, molecular mimicry between *Borrelia* antigens and myelin proteins has been hypothesized, although direct causal evidence remains limited. Experimental data have also suggested potential molecular mimicry between Borrelia flagellin and neuronal antigens, including heat shock protein 60 (HSP60). A monoclonal anti-flagellin antibody (H9724) has been shown to alter dendritic outgrowth in neuroblastoma models; however, the clinical relevance of these findings remains uncertain [34]. For accurate attribution, current guidelines emphasize the necessity of CSF pleocytosis and intrathecal *Borrelia*-specific antibody synthesis, as serum antibodies alone lack specificity in endemic regions [30,31,33].

Beyond MS-like phenotypes, autoimmune encephalomyelitis represents another immune-mediated manifestation affecting the brain and spinal cord, classically exemplified by acute disseminated encephalomyelitis (ADEM). Clinically, these syndromes present with subacute encephalopathy, altered mental status, seizures, multifocal neurological deficits, ataxia, and diffuse MRI abnormalities involving both white and gray matter. In the context of Lyme disease, such presentations are rare but have been described as post-infectious immune phenomena following confirmed LNB [30,32,35].

The proposed immunopathogenesis involves infection-triggered immune priming, whereby intense CNS inflammation during LNB leads to exposure of self-antigens and subsequent activation of autoreactive T and B lymphocytes. Epitope spreading and dysregulated adaptive immune responses may then sustain parenchymal inflammation even after bacterial clearance. This mechanism is supported by cases in which neurological deterioration persists despite appropriate antibiotic therapy but improves following immunomodulatory treatment, suggesting a transition from infection-driven to immune-mediated pathology [32,35].

In a related but distinct context, autoimmune encephalitis encompasses a group of disorders mediated by antibodies targeting neuronal surface or synaptic proteins, such as the N-methyl-D-aspartate receptor (NMDAR). Clinical manifestations include rapidly progressive cognitive decline, psychiatric symptoms, seizures, dyskinesias, speech disturbance, and autonomic instability. In rare instances, autoimmune encephalitis has been reported in temporal association with LNB [36].

The hypothesized link centers on infection-induced immune dysregulation, whereby severe intrathecal inflammation, BBB disruption, and antigen presentation may facilitate the emergence of pathogenic neuronal autoantibodies through molecular mimicry or loss of immune tolerance. However, cohort studies indicate that neuronal autoantibodies are not commonly detected in LNB, underscoring that this association is exceptional and not typical and should be considered a rare secondary immune complication instead of a defining manifestation of Lyme disease [36,37].

Chronic inflammatory meningoencephalitis associated with LD represents another manifestation of sustained immune activation within the CNS and presents with progressive cognitive decline, behavioral changes, chronic headache, fatigue, gait disturbance, and focal neurological deficits, often evolving over months or years. Unlike acute LNB, this phenotype reflects prolonged immune activation, sometimes due to delayed diagnosis or incomplete treatment, with MRI findings frequently nonspecific and CSF typically demonstrating persistent lymphocytic inflammation [32,38].

Immunopathologically, sustained B-cell recruitment driven by CXCL13 and ongoing T-cell activation may maintain a chronic inflammatory state, resulting in neuronal dysfunction and potentially reversible neurodegeneration. These findings further support the concept that long-term neurological impairment is driven primarily by immune-mediated mechanisms [32,38].

Among the most severe immune-mediated CNS complications, cerebral vasculitis represents a rare but devastating manifestation of LNB, presenting with ischemic stroke, transient ischemic attacks, focal neurological deficits, and severe headache. Neuroimaging often reveals multifocal infarcts or vessel stenosis, while CSF shows inflammatory changes consistent with LNB. The underlying mechanism involves immune-mediated vascular inflammation driven by cytokine-induced endothelial activation and leukocyte infiltration of cerebral vessel walls, leading to vessel narrowing, thrombosis, and cerebral ischemia. In selected cases, combined antimicrobial and anti-inflammatory therapy may therefore be required [39,40].

Finally, persistent cognitive complaints following Lyme disease are most commonly discussed under post-treatment Lyme disease syndrome (PTLDS), defined by new or ongoing symptoms lasting at least six months after recommended antibiotic therapy in patients with documented Lyme disease and without evidence of an alternative diagnosis. The cognitive phenotype is typically described as “brain fog,” encompassing slowed processing speed, impaired attention and concentration, reduced working memory, word-finding difficulty, mental fatigue, and reduced multitasking capacity. These symptoms frequently coexist with fatigue, sleep disturbance, pain, and mood symptoms that may amplify perceived cognitive dysfunction.

Objective neuropsychological testing demonstrates heterogeneous results across cohorts, with some showing measurable deficits and others largely normal performance, indicating that PTLDS represents a syndrome with variable objective correlates not a single uniform cognitive disorder [41,42,43].

Within this framework, PTLDS is generally considered a post-infectious, immune-amplified neurophysiologic state. Proposed mechanisms include persistent immune activation, inflammatory signaling following spirochetal clearance, microglial priming, and neuroinflammation-adjacent processes that affect neural network efficiency and sickness-behavior pathways. Reviews emphasize that the inflammatory response in acute Lyme disease and LNB is prominently B-cell driven, including CXCL13-mediated recruitment, and that in a subset of patients, immune dysregulation may persist after the inciting infection, producing long-lasting neurological symptoms despite microbiologic cure. In addition, it is possible that initial infection and associated inflammatory responses induce persistent alterations in neural network function that do not require ongoing immune activation, reflecting long-term neurophysiologic changes following the acute disease phase [41,42,44].

##### Peripheral Nervous System (PNS) Autoimmune Mediated Manifestations Associated with LD

PNS involvement in LD most often arises in the setting of neuroinflammatory responses accompanying Bbsl infection, classically manifesting as radiculoneuritis or cranial neuropathies. Beyond these well-recognized presentations, accumulating clinical observations describe a broader range of peripheral neuropathic phenotypes in which immune-mediated mechanisms predominate, either persisting after microbiologic resolution or overlapping with inflammatory neuropathy syndromes.

These processes are thought to reflect infection-triggered immune activation involving spirochetal lipoprotein-driven innate immune signaling, downstream cytokine and chemokine cascades, disruption of the blood–nerve barrier, and maladaptive adaptive immune responses, collectively facilitating secondary nerve injury and, in selected cases, autoimmune-like neuropathic patterns [30,31,45].

Within this context, chronic inflammatory demyelinating polyneuropathy (CIDP)-like associated with LD has been described as a chronic or subacute sensorimotor disorder marked by symmetrical weakness, sensory loss, areflexia, and gait disturbance evolving over more than eight weeks, with electrophysiologic findings suggestive of demyelination. While CIDP is a well-characterized autoimmune disorder, its association with LD is rare and supported primarily by case reports and limited observational data.

In LNB, demyelinating features are more plausibly interpreted as the consequence of sustained immune activity within peripheral nerves and nerve roots, producing a CIDP-like phenotype rather than constituting true primary CIDP. Proposed immunopathologic mechanisms include macrophage-driven myelin injury, T-cell-mediated inflammation, and antibody-associated damage to myelin or nodal regions, potentially initiated by infection-related immune dysregulation [31,45,46,47]. Isolated serum *Borrelia* seropositivity does not establish causation, underscoring the importance of distinguishing immune-mediated neuropathy from infectious radiculoneuritis and idiopathic CIDP [28,45,46,47].

In contrast, inflammatory axonal neuropathy is characterized by distal sensory deficits, paresthesias, neuropathic pain, and variable motor involvement, typically demonstrating a length-dependent pattern with electrophysiologic evidence of axonal loss as opposed to demyelination. Although axonal neuropathy has historically been proposed as a late manifestation of LD, its status as a distinct Lyme-associated condition remains debated. Many reported cases are limited by confounding comorbidities or insufficient confirmation of LNB.

In contemporary interpretations, axonal injury occurring in temporal association with LD is more often attributed to immune-mediated neurotoxicity, cytokine-associated inflammation, or microvascular dysfunction of axons, and should therefore be regarded as a hypothesized but unproven immune-mediated association requiring careful exclusion of alternative causes [47,48].

Another peripheral phenotype increasingly discussed in post-Lyme cohorts is small fiber neuropathy, which selectively affects thinly myelinated Aδ fibers and unmyelinated C fibers responsible for nociceptive and autonomic signaling. In patients with PTLDS, this condition is characterized by reduced intraepidermal nerve fiber density and abnormal autonomic function testing despite preserved large-fiber conduction.

The underlying mechanism is thought to involve post-infectious immune imbalance and persistent neuroinflammatory signaling that preferentially targets small fibers, providing a mechanistic explanation for chronic sensory and autonomic complaints following microbiologic cure. The preferential involvement of thinly myelinated Aδ fibers and unmyelinated C fibers may reflect their increased vulnerability to immune-mediated injury, related to their limited myelin protection, superficial anatomical distribution, and heightened sensitivity to cytokine-mediated and microvascular inflammatory processes [49].

Closely related to small fiber involvement, autonomic neuropathy associated with LD presents with orthostatic intolerance, palpitations, presyncope or syncope, thermoregulatory instability, gastrointestinal dysmotility, bladder dysfunction, and reduced exercise tolerance, often accompanied by fatigue and cognitive symptoms. Dysautonomia has emerged as a recognized component of PTLDS and shares features with other post-infectious autonomic disorders.

Proposed immunopathologic contributors include immune-mediated injury to autonomic fibers, autoantibody production, persistent neuroimmune activation, and impaired vasomotor regulation. While LD is considered a plausible precipitating factor, available evidence supports an associative relationship, reinforcing the need for thorough evaluation and caution against attributing symptoms to persistent infection [50].

Radiculopathy occupies a central position within the PNS manifestations of LNB and results from inflammatory involvement of nerve roots and adjacent meninges, producing severe radicular pain, sensory disturbances, and variable motor deficits, typically accompanied by cerebrospinal fluid lymphocytic pleocytosis and intrathecal *Borrelia*-specific antibody synthesis [30,31].

Although initially driven by infection-associated inflammation, the intensity and duration of nerve root involvement may extend beyond bacterial clearance, and in a subset of patients persistent radicular symptoms reflect ongoing immune-mediated dysfunction [31,51].

In uncommon instances, this inflammatory process may extend to the brachial or lumbosacral plexus, leading to plexopathy-like syndromes characterized by severe pain followed by weakness and sensory loss.

Such presentations are best interpreted as atypical immune-inflammatory extensions of radiculoneuritis not primary plexopathies, with presumed mechanisms involving infection-triggered inflammatory or microvasculitic injury to plexus nerves.

Given the limited and heterogeneous evidence, plexus involvement should be viewed as a rare and nonspecific component within the broader spectrum of immune-mediated peripheral nervous system manifestations associated with LD [30,31,45].

**Figure 2:**
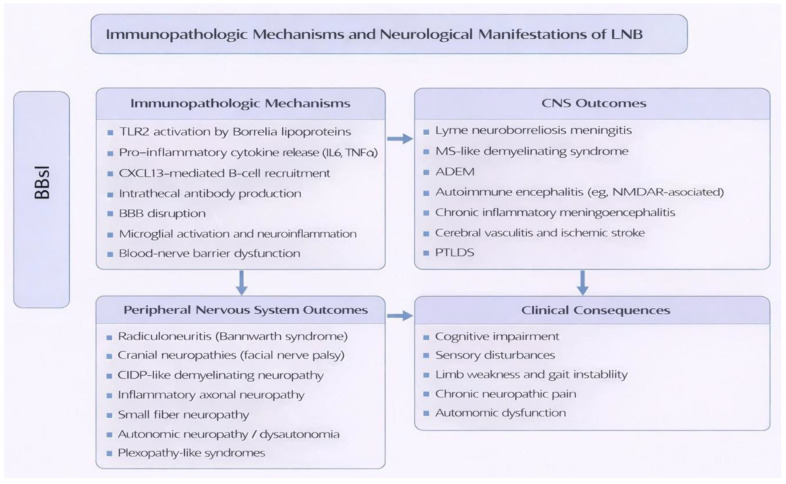
Immunopathologic mechanisms and neurological manifestations associated with LNB. ADEM—Acute Disseminated Encephalomyelitis; NMDAR—N-Methyl-D-Aspartate Receptor; CNS—Central Nervous System; PNS—Peripheral Nervous System; TLR2—Toll-Like Receptor 2; IL—Interleukin; TNF—Tumor Necrosis Factor; PTLDS—Post-Treatment Lyme Disease Syndrome; CIDP—Chronic Inflammatory Demyelinating Polyneuropathy; LNB—Lyme Neuroborreliosis; Bbsl—*Borrelia burgdorferi sensu lato*; BBB—Blood–Brain Barrier; CXCL13—C-X-C Motif Chemokine Ligand 13; MS—Multiple Sclerosis.

A schematic overview of the immunopathologic mechanisms and resulting CNS and PNS manifestations of LNB is presented in Figure 2.

#### 3.2.2. Involvement of the Musculoskeletal System

**Figure 3:**
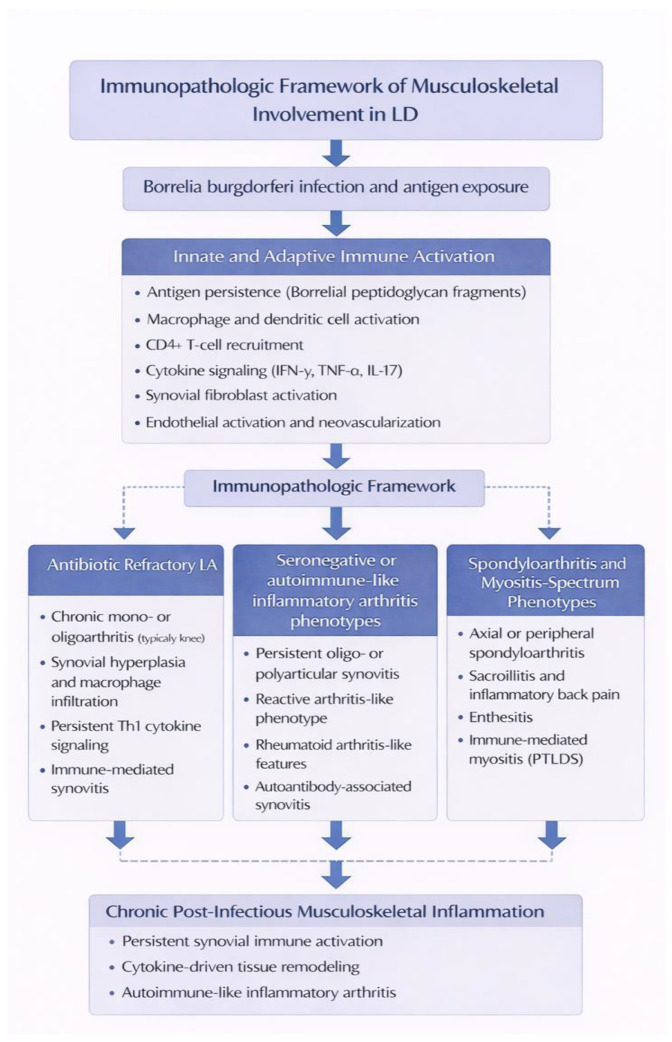
Immunopathologic Framework of Musculoskeletal Involvement in LD. Th1—T helper 1; IL—Interleukin; TNF—Tumor Necrosis Factor; IFN—Interferon; LD—Lyme Disease; LA—Lyme Arthritis; PTLDS—Post-Treatment Lyme Disease Syndrome.

Musculoskeletal involvement in LD reflects infection-triggered immune dysregulation that may persist beyond microbial clearance. The immunopathologic mechanisms and the spectrum of resulting inflammatory phenotypes are summarized in Figure 3.

The musculoskeletal involvement of Lyme disease reflects, in part, the tissue tropism of Bbsl, which preferentially localizes to connective tissues, including synovium, where interactions with extracellular matrix components and host immune responses facilitate persistent inflammation.

Acute Lyme arthritis represents a common late manifestation of untreated or inadequately treated LD and typically presents as monoarthritis or oligoarthritis, most frequently affecting large joints such as the knee. The condition is characterized by marked joint swelling with relatively mild pain and limited systemic symptoms. Pathophysiologically, acute arthritis reflects a combination of spirochetal presence within the synovium and a robust host immune response, with inflammatory cytokine production and immune cell infiltration contributing to synovial inflammation, in the context of Bbsl tropism for synovial and connective tissues.

##### Antibiotic-Refractory LA

Antibiotic-refractory LA is characterized by persistent or recurrent inflammatory mono- or oligoarthritis, most commonly affecting the knee, that continues for months or even years despite appropriate and microbiologically adequate antibiotic therapy and in the absence of evidence for ongoing *Borrelia* infection. This clinical phenotype is now widely interpreted as a post-infectious, immune-mediated condition instead of a consequence of antimicrobial treatment failure [7,8,9]. Immunopathologic investigations consistently demonstrate that synovial inflammation in antibiotic-refractory LA is sustained by ongoing immune activation.

Synovial tissue, consistent with Bbsl tropism for connective and synovial tissues, typically shows dense infiltration by CD4^+^ T lymphocytes and activated macrophages, along with marked synovial hyperplasia, neoangiogenesis, and fibroblast activation. The local cytokine environment is predominantly Th1-skewed, with prominent expression of IFN-γ and tumor necrosis factor-α, which together drive macrophage activation, synoviocyte proliferation, and progressive matrix remodeling within the joint [7,8,52].

A defining mechanistic feature of this condition is persistent antigenic stimulation in the absence of viable organisms, as discussed in Section 3.1.5. *Borrelial* peptidoglycan and residual antigenic debris retained within synovial tissue consistent with preferential localization of Bbsl components in these tissues continue to engage innate and adaptive immune pathways, thereby sustaining inflammation long after bacterial eradication. This antigen-driven immune activation provides a mechanistic bridge between infection-associated inflammation and autoimmune-like pathology without implying ongoing infection [21,22].

Host genetic factors play a critical role in determining progression to an antibiotic-refractory course. Strong associations have been identified between this phenotype and specific HLA-DR alleles capable of efficiently presenting immunodominant *Borrelia* peptides to CD4^+^ T cells. These HLA-restricted responses favor prolonged T-cell activation and impaired contraction of adaptive immune responses, offering a mechanistic explanation for the persistence of synovial inflammation in genetically susceptible individuals [25,53].

With ongoing antigen presentation and sustained inflammatory signaling, immune responses may broaden over time through epitope spreading toward host joint antigens. Immunologic profiling studies have demonstrated expansion of autoreactive T-cell populations and recognition of synovial autoantigens during disease progression, consistent with a transition from infection-triggered inflammation to self-perpetuating immune-mediated arthritis [27,29]. At the same time, regulatory mechanisms—including regulatory T-cell-mediated suppression—appear insufficient to re-establish immune homeostasis in this setting, further reinforcing chronic inflammation [18].

Taken together, antibiotic-refractory Lyme arthritis represents a prototypical example of infection-triggered, antigen-driven immune-mediated disease, in which microbial exposure initiates a pathogenic immune response that persists after pathogen clearance. This model provides a conceptual framework for understanding other post-infectious inflammatory phenotypes associated with LD and highlights the need for therapeutic strategies that extend beyond antimicrobial therapy alone [8,20,52].

##### Seronegative and Autoimmune-like Inflammatory Arthritides Following LD

Seronegative inflammatory arthritis following LD, defined in this context by the absence of rheumatoid factor and anti-cyclic citrullinated peptide antibodies (and not by the absence of *Borrelia*-specific antibodies), manifests as persistent oligoarticular or polyarticular synovitis, with clinical features consistent with reactive or seronegative autoimmune arthritis, not classic Lyme monoarthritis [54,55,56].

Proposed mechanisms include bystander activation of autoreactive lymphocytes, sustained activation of macrophages and fibroblast-like synoviocytes, and cytokine-driven amplification of synovial inflammation initially initiated during acute infection [54,55,56]. In this setting, *Borrelia* exposure functions primarily as an inflammatory trigger that lowers the threshold for persistent immune activation in predisposed hosts.

Along a related clinical spectrum, rheumatoid arthritis-like disease temporally associated with LB manifests as symmetric inflammatory polyarthritis involving small joints, accompanied by prolonged morning stiffness and chronic synovitis. Immunologically, these cases align more closely with infection-triggered acceleration or unmasking of pre-existing autoimmune disease [55,56].

Infection-associated cytokine release and enhanced antigen presentation may promote the emergence or amplification of autoantibody-mediated synovial inflammation, including rheumatoid factor and anti-CCP antibodies, particularly in genetically susceptible individuals, supporting the interpretation of LD as an environmental precipitant, not an etiologic agent of rheumatoid arthritis.

##### Spondyloarthritis- and Myositis-Spectrum Phenotypes

Beyond RA-like patterns, rare seronegative spondyloarthritis phenotypes reported after LD include inflammatory back pain, peripheral arthritis, and psoriatic arthritis-like presentations. From an immunopathologic standpoint, these phenotypes are driven by innate–adaptive immune interactions, with prominent involvement of IL-17/IL-23 signaling pathways and immune activation at entheses.

LD has been hypothesized to act as an inflammatory stimulus that amplifies these pathways in genetically predisposed individuals, facilitating transition from transient post-infectious inflammation to chronic spondyloarthritis-type disease; however, available evidence is limited and largely derived from isolated reports, and this relationship is best framed as immune triggering [55,56].

Inflammatory sacroiliitis developing after LD represents a related axial manifestation, presenting with inflammatory buttock and low back pain and MRI evidence of active sacroiliac inflammation, including non-radiographic axial spondyloarthritis phenotypes. The immunologic framework mirrors that of axial spondyloarthritis, characterized by entheseal immune activation, cytokine-driven inflammation, and bone marrow edema, with LD potentially contributing by augmenting systemic inflammatory signaling and lowering the threshold for axial immune activation in susceptible individuals, although current data remain limited to case-based observations [57].

Enthesitis occurring in post-Lyme inflammatory arthritis reflects immune-mediated inflammation at tendon and ligament insertions and is driven by innate immune activation at mechanically stressed entheses, local cytokine production, and recruitment of inflammatory cells that sustain tissue injury [55,56,57]. The involvement of entheses may relate to the preferential localization of inflammatory responses in mechanically stressed connective tissues, which are particularly susceptible to cytokine-mediated immune activation in the context of Bbsl-associated immune dysregulation. In Lyme-associated cases, enthesitis is most appropriately interpreted as part of a broader seronegative inflammatory arthritis spectrum triggered by infection-induced immune dysregulation [55,56,57].

Inflammatory involvement of skeletal muscle represents a further, uncommon extension of post-Lyme immune-mediated musculoskeletal disease. Inflammatory myositis associated with LD includes focal or diffuse muscle inflammation, with reported cases demonstrating muscle edema and inflammatory infiltrates on imaging and variable responsiveness to antimicrobial therapy.

Immunologically, muscle involvement is attributed to infection-associated immune activation, with cytokine-mediated myocyte dysfunction and immune-cell infiltration contributing to muscle inflammation, supporting classification as a rare immune-mediated complication of LD [58,59]. This pattern may reflect the vulnerability of muscle tissue to systemic inflammatory signaling and immune-mediated injury, not direct pathogen tropism.

Polymyositis-like and dermatomyositis-spectrum phenotypes temporally associated with LD further support a post-infectious autoimmunity model, in which immune activation during infection promotes loss of tolerance to muscle antigens. Detection of myositis-associated autoantibodies in a subset of patients evaluated for PTLDS suggests that LD-induced immune dysregulation may intersect with established autoimmune myopathy pathways, reinforcing immune triggering [60,61].

Finally, chronic inflammatory myalgias following LD are most frequently observed within PTLDS and present as persistent diffuse muscle pain, stiffness, and exercise intolerance. These symptoms are immunologically attributed to ongoing low-grade immune activation and neuroimmune dysregulation, including cytokine-mediated sickness behavior and central sensitization, instead of active musculoskeletal infection, reflecting a post-infectious inflammatory state with heterogeneous objective correlates and limited response to prolonged antimicrobial therapy [41].

#### 3.2.3. Immunopathologic Framework of Cardiovascular Involvement in LD

**Figure 4:**
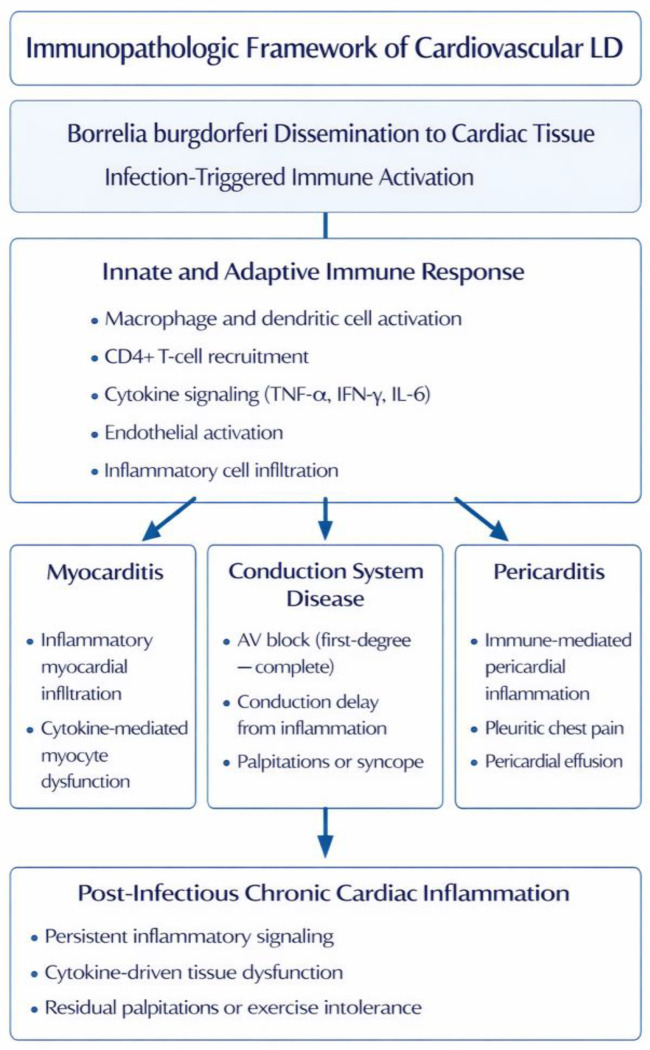
Immunopathologic framework of cardiovascular involvement in LD. LD—Lyme disease; AV—atrioventricular; IFN-γ—interferon gamma; IL-6—interleukin-6; TNF-α—tumor necrosis factor alpha.

Cardiovascular involvement in LD, Lyme carditis arises following dissemination of Bbsl to cardiac tissue and is characterized by a prominent inflammatory host response. Although direct infection initiates cardiac involvement, multiple lines of experimental and clinical evidence indicate that immune-mediated amplification of tissue injury accounts for the severity, variability, and frequent reversibility of cardiac manifestations. This paradigm aligns with broader infection-triggered immunopathogenic mechanisms described earlier, including cytokine-driven tissue dysfunction, endothelial activation, and sustained inflammatory signaling (Figure 4) [62,63,64]. The apparent cardiac tissue involvement likely reflects not only dissemination of Bbsl but also the susceptibility of cardiac tissue particularly the conduction system and myocardium to inflammation-mediated dysfunction, given their sensitivity to cytokine signaling, immune-cell infiltration, and disruption of electrophysiologic homeostasis.

Myocardial involvement represents a central component of this spectrum. Lyme-associated myocarditis is characterized by inflammatory infiltration of the myocardium and cytokine-mediated myocyte dysfunction, producing chest pain, dyspnea, exercise intolerance, palpitations, and biomarker elevation, with supportive imaging and histopathologic findings [61,63,65]. Importantly, the degree of myocardial inflammation and functional impairment may be disproportionate to detectable spirochetal burden, supporting an immune-mediated mechanism of injury. Innate and adaptive immune activation, together with endothelial and stromal cell responses, provides a biologic basis for myocardial dysfunction that may transiently persist after microbial clearance. Myocardial inflammation frequently overlaps with conduction system involvement, reflecting a shared inflammatory substrate within cardiac tissue.

Inflammatory involvement of the pericardium represents a less common but recognized manifestation within the Lyme carditis spectrum. Pericarditis typically presents with pleuritic chest pain, pericardial rub, electrocardiographic changes, pericardial effusion, and elevated inflammatory markers. Pericardial inflammation is best interpreted as immune-mediated, driven by cytokine- and chemokine-dependent recruitment of inflammatory cells [62,63]. The frequent coexistence of pericarditis with myocarditis or conduction abnormalities supports the concept of a pancarditis phenotype sustained by infection-associated immune activation.

Conduction system disease constitutes the hallmark manifestation of Lyme carditis and ranges from first-degree atrioventricular (AV) block to complete heart block, often with rapid fluctuation in severity. These disturbances result from inflammatory involvement of the cardiac conduction system, including immune-cell infiltration and cytokine-mediated slowing of electrical conduction [62,63,64,65,66,67]. The dynamic nature and high reversibility of AV block strongly support a predominantly inflammatory mechanism rather than fixed structural injury; however, the rapid improvement observed following antibiotic therapy also suggests that local infection and its resolution may contribute to the reduction in inflammation and recovery of conduction function. Although conduction abnormalities typically resolve following appropriate therapy, residual conduction delay or symptoms during convalescence are generally attributed to ongoing inflammatory remodeling.

Arrhythmias in Lyme carditis are most commonly described in association with myocarditis, pericarditis, or conduction system inflammation and may include supraventricular arrhythmias, ventricular ectopy, and symptomatic palpitations, although systematic data remain limited and are largely derived from case series and observational reports [50,62]. In post-infectious settings, persistent palpitations, exercise intolerance, or orthostatic symptoms may reflect overlapping myocardial and autonomic immune-mediated dysfunction, consistent with neuroimmune mechanisms described elsewhere, as opposed to fixed arrhythmogenic foci.

In contrast, non-infectious inflammatory endocarditis represents a speculative and poorly substantiated entity in LD. While rare cases of infective Lyme endocarditis have been documented with microbiologic confirmation, a distinct non-infectious, immune-mediated endocarditis triggered by *Borrelia* remains theoretical and should be regarded as exceptionally rare or unproven [66,68,69]. As such, this entity should be interpreted cautiously and only after exclusion of alternative systemic inflammatory, autoimmune, or thrombotic conditions.

#### 3.2.4. Immunopathologic Framework of Cutaneous Involvement in LD

**Figure 5:**
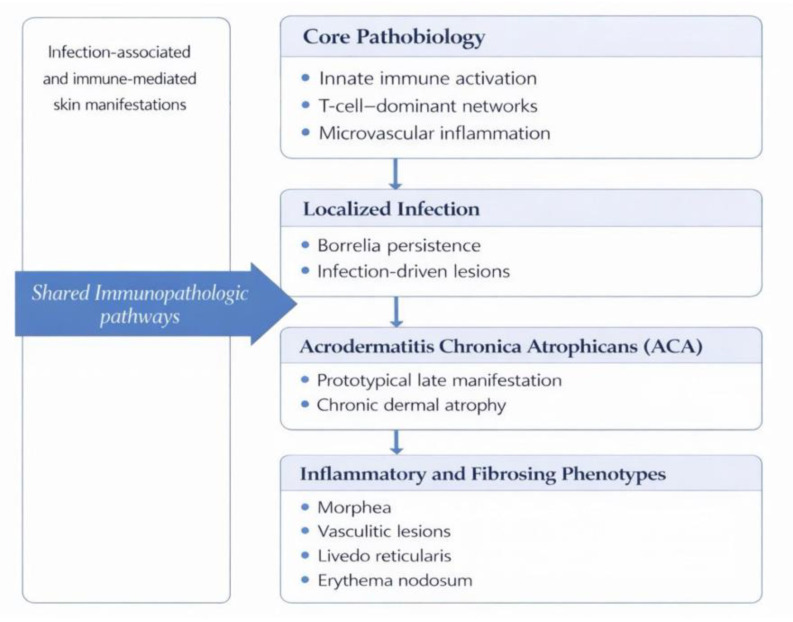
Cutaneous Involvment in LD. LD—Lyme Disease; ACA—Acrodermatitis Chronica Atrophicans.

Cutaneous involvement in LD encompasses both infection-associated skin manifestations and a broader spectrum of inflammatory dermatoses best explained by infection-triggered immune dysregulation. While certain phenotypes reflect localized persistence of *Borrelia* within the skin, many cutaneous manifestations—particularly those that persist, evolve, or arise after appropriate antimicrobial therapy—are more plausibly driven by immune-mediated mechanisms. Across this spectrum, shared immunopathological features include *Borrelia*-induced innate immune activation, T-cell-dominant chemokine networks, endothelial and microvascular inflammation, and, in chronic settings, fibroblast activation with extracellular matrix remodeling (Figure 5) [70,71,72].

Erythema migrans (EM) represents the earliest and most characteristic cutaneous manifestation of LD and reflects localized infection at the site of tick inoculation. Histopathologically, EM is associated with a perivascular and interstitial inflammatory infiltrate composed of lymphocytes, macrophages, and plasma cells, accompanied by local cytokine and chemokine production. While viable spirochetes may be detected within the lesion, the extent of inflammation is often disproportionate to bacterial burden, supporting a significant contribution of host immune responses even in early disease. EM therefore represents a transitional phenotype linking direct infection with early immune activation [73].

Acrodermatitis chronica atrophicans (ACA) represents the most clearly established late cutaneous manifestation of European LD and provides a prototypical example of chronic immune-mediated skin pathology. Immunopathologic studies demonstrate persistent lymphoplasmacytic infiltration and a strong T-cell-associated chemokine signature within affected skin, including high expression of interferon-inducible chemokines such as CXCL9 and CXCL10 [71,74,75]. These findings support a model in which sustained immune activation, drives progressive tissue remodeling, ultimately resulting in dermal atrophy, loss of adnexal structures, and associated neuropathic symptoms.

Other fibrosing or inflammatory dermatoses temporally associated with LD are best interpreted through post-infectious immune frameworks. Morphea (localized scleroderma), a fibrosing inflammatory condition characterized by T-cell and macrophage activation, cytokine-driven fibroblast stimulation, and excessive extracellular matrix deposition, has been extensively investigated as a potential *Borrelia*-associated phenotype. However, large molecular studies have failed to demonstrate consistent detection of *Borrelia* DNA within morphea lesions, indicating that most cases are not attributable to persistent infection [76,77]. When temporal associations are observed, morphea is best framed as an uncommon immune-mediated phenotype potentially triggered by infection-induced immune dysregulation [76,77,78].

Vascular inflammatory phenotypes further illustrate the capacity of *Borrelia*-triggered immune activation to produce secondary cutaneous pathology. Cutaneous vasculitides reported in association with LD are most often interpreted as immune-complex-mediated or cytokine-driven small-vessel inflammation. Leukocytoclastic vasculitis and immunoglobulin (Ig) A vasculitis-like presentations reflect immune activation with complement-dependent vascular injury in the context of systemic inflammation and antigen exposure [79,80,81]. Similarly, livedo reticularis and livedo racemosa are best understood as manifestations of immune-mediated endothelial dysfunction, vasospasm, or microvascular injury, although attribution to Lyme disease should remain conservative given the broad differential diagnosis [82].

Inflammatory panniculitides further support an infection-triggered immune model. Erythema nodosum and other panniculitic patterns reported in association with LD are mediated by hypersensitivity-type immune responses within subcutaneous fat and reflect systemic immune activation [83,84,85,86]. These entities align with well-established paradigms of infection-induced immune dysregulation producing secondary inflammatory skin disease.

Finally, autoimmune-predominant dermatoses such as lichen sclerosus have been explored as potential post-infectious phenomena in selected populations. Although characterized by T-cell-mediated inflammation and progressive tissue sclerosis, evidence supporting *Borrelia* as a consistent trigger remains inconsistent and geographically variable. Current data support interpretation of *Borrelia* as a biologically plausible but unproven immune trigger in rare cases instead of a generalizable cause [86,87].

Collectively, cutaneous manifestations of LD illustrate how localized infection can initiate immune pathways that evolve into persistent inflammatory, vascular, or fibrosing skin phenotypes. These processes reflect broader principles of infection-triggered immune dysregulation and provide a conceptual bridge to other immune-mediated complications of LD discussed in subsequent sections.

#### 3.2.5. Immunopathologic Framework of Hematologic Involvement in LD

Hematologic involvement in LD is uncommon and typically mild; however, a small number of immune-mediated cytopenias have been reported as rare complications occurring during acute infection or as post-infectious immune phenomena. These manifestations are best interpreted through infection-triggered immune dysregulation. Proposed immunopathologic mechanisms include molecular mimicry, polyclonal B-cell activation, immune-complex formation, complement activation, cytokine-driven immune amplification, and enhanced phagocytic clearance of peripheral blood elements [2,3,23,24,73]. Given the rarity of these entities and the substantial risk of confounding, hematologic immune manifestations attributed to LD should be interpreted conservatively and only after exclusion of alternative causes, including other tick-borne coinfections, medication effects, viral illness, and primary hematologic disorders [56,73].

Among reported phenotypes, immune thrombocytopenia represents the most frequently described immune-mediated hematologic complication temporally associated with LD. Case reports and small series describe isolated or recurrent thrombocytopenia occurring in conjunction with acute *Borrelia* infection, with clinical severity ranging from asymptomatic laboratory abnormalities to mucocutaneous bleeding [88,89]. The underlying immunopathology is consistent with antibody-mediated platelet opsonization and Fcγ receptor-dependent clearance by splenic and hepatic macrophages, potentially amplified by infection-induced innate immune activation and inflammatory cytokine signaling. Improvement in platelet counts following antimicrobial therapy and, in some cases, immunomodulatory treatment supports an immune-amplified mechanism and a direct suppression of hematopoiesis [4,88].

Autoimmune hemolytic anemia has been reported far less frequently and is primarily documented in isolated case reports, occasionally occurring alongside thrombocytopenia. These cases are most consistent with warm-type autoimmune hemolytic anemia mediated by IgG autoantibodies and extravascular erythrocyte destruction [5,73]. In the context of LD, infection-triggered immune activation may promote breakdown of tolerance and autoantibody generation in susceptible individuals. Given its extreme rarity, autoimmune hemolytic anemia should be regarded as an exceptional immune complication and not a characteristic hematologic manifestation.

Autoimmune leukopenia or neutropenia has been reported only sporadically in association with LD and remains poorly characterized. Mechanistically, such presentations would plausibly involve anti-leukocyte antibodies and immune-mediated peripheral destruction facilitated by systemic immune activation. In clinical and academic contexts, leukopenia observed in patients with suspected or confirmed LD requires careful evaluation for alternative explanations, including coinfections, medication effects, viral illnesses, and primary marrow pathology. Attribution to Lyme-triggered autoimmunity should therefore be made cautiously and only after exclusion of more common causes [73,89].

Immune-mediated pancytopenia represents an exceptionally rare and controversial association. Pancytopenia following tick exposure is far more commonly attributable to alternative infectious or hematologic conditions. When discussed in relation to LD, the most defensible immunopathologic framework involves infection-triggered systemic immune activation leading to immune-mediated peripheral destruction and/or cytokine-driven suppression of hematopoiesis without evidence of direct marrow infection [73,89,90]. Given the limited evidence base and high risk of misattribution, this entity should be presented as a rare and speculative immune complication requiring comprehensive hematologic evaluation prior to causal linkage.

### 3.3. Controversies, Limitations, and Areas of Uncertainty

Despite growing evidence that immune-mediated mechanisms contribute to selected manifestations of LD, many proposed associations between Bbsl infection and systemic autoimmune or immune-mediated disorders remain controversial. A substantial proportion of reported links derive from case reports, small observational cohorts, or temporal associations, which limit causal inference. As a result, careful distinction is required between biologically plausible immune triggering, transient infection-associated immune activation, coincidental disease overlap, diagnostic misattribution, and true post-infectious immune pathology. Importantly, the coexistence of documented LD and a subsequent immune-mediated condition does not establish causality, as such associations may reflect coincidence, not a pathogenic relationship.

Systemic autoimmune disease-like phenotypes temporally associated with LD, including systemic lupus erythematosus-like presentations, have been described. These reports often include inflammatory arthritis, cutaneous findings, cytopenia, and transient autoantibody positivity. The most plausible immunopathologic explanation involves infection-driven polyclonal B-cell activation and transient loss of immune tolerance with no evidence induction of sustained systemic autoimmunity. Current evidence does not support LD as a causal factor for systemic lupus erythematosus, and most reported cases are best interpreted as coincidental coexistence or diagnostic overlap as opposed to post-infectious autoimmune disease [56,71,91,92]. Similarly, Sjögren syndrome-like features reported following LD, such as sicca symptoms and fatigue, occur against a high background prevalence in the general population and should be framed as nonspecific immune overlap or triggering in predisposed individuals rather than defined Lyme-associated autoimmune syndromes [93].

Endocrine and gastrointestinal autoimmune associations remain weakly supported. Autoimmune thyroiditis has been reported during or after disseminated LD, and infection-triggered immune activation provides a biologically plausible mechanism for transient thyroid immune dysfunction. However, autoimmune thyroid disease is common, and available data do not establish a causal relationship with *Borrelia* infection [94,95]. Proposed links between LD and celiac disease-like or inflammatory bowel disease-like syndromes are similarly unsupported, with epidemiologic studies failing to demonstrate a meaningful increase in risk. Gastrointestinal symptoms are more plausibly explained by systemic inflammation, immune activation, or unrelated pathology with limited support for true autoimmune disease triggered by LD [96,97].

Reports of systemic vasculitis following LD are rare and heterogeneous. Although immune-complex deposition, endothelial activation, and dysregulated adaptive immunity represent biologically plausible mechanisms, supporting evidence is limited and frequently confounded. Systemic vasculitis should therefore be regarded as an uncommon and controversial overlap rather not a characteristic immune-mediated manifestation of LD [98,99]. Transient antiphospholipid antibody positivity has also been documented during acute infections, including LD, reflecting nonspecific immune activation instead of true antiphospholipid syndrome. LD is best positioned as a potential trigger of transient serologic abnormalities without clinical significance in most cases [100].

Ocular and renal immune-mediated manifestations further illustrate the need for caution in attribution. Inflammatory ocular phenotypes such as uveitis and retinal vasculitis have been reported in temporal association with LD. Still, these cases are uncommon and require rigorous exclusion of alternative infectious and autoimmune etiologies before causal linkage is considered [101,102]. Similarly, immune-mediated glomerulonephritis, including immune-complex-mediated patterns, has been described rarely in the setting of LD. While circulating immune complexes and complement activation provide a plausible mechanistic framework, current evidence does not support Lyme disease as a common or primary driver of renal immune pathology [103,104].

LD can plausibly trigger transient immune dysregulation and inflammatory overlap syndromes, but progression to bona fide systemic autoimmune disease remains unproven in most settings. Rigorous phenotyping, exclusion of confounders, and avoidance of over-attribution are essential to accurately define the boundaries of immune-mediated pathology associated with Bbsl infection.

### 3.4. Adjunctive Immunological Biomarkers in Lyme-Associated Manifestations

Standard two-tier serologic testing confirms exposure to Bbsl but cannot reliably distinguish active infection from post-infectious inflammatory sequelae. Among currently available adjunctive biomarkers, only a limited subset demonstrates relative disease specificity within defined organ systems (Table 2).

CSF CXCL13 is the most sensitive biomarker for early LNB and has demonstrated high diagnostic performance in patients with acute CNS involvement [32,40,105]. Intrathecal synthesis of *Borrelia*-specific antibodies, assessed by antibody index calculation, remains the reference laboratory standard for confirming NB [32]. These markers support the presence of intrathecal inflammatory activity but do not determine pathogen viability.

In antibiotic-refractory LA, borrelial peptidoglycan has been identified in synovial fluid despite the absence of culturable organisms [21]. Borrelial peptidoglycan fragments may persist in synovial tissue and sustain innate immune activation, contributing to chronic synovial inflammation. Detection of this persistent antigen represents one of the most specific molecular findings in chronic Lyme-associated inflammatory arthritis and may assist in distinguishing immune-mediated persistence from active infection.

In contrast, no validated disease-specific molecular biomarkers have been established for Lyme-associated cardiac or hematologic manifestations. In suspected Lyme carditis, cardiac troponins and electrocardiographic abnormalities may indicate myocardial involvement but lack specificity for Bbs*l* infection [91]. Similarly, mild cytopenias observed in disseminated disease are nonspecific findings and are more frequently associated with systemic inflammation or tick-borne co-infections [90,106]. At present, these parameters remain supportive clinical indicators and not diagnostic biomarkers.

## 4. Conclusions

LD should be understood not only as an infectious disease but also as a condition involving complex host immune responses. In this review, we examined experimental, translational, and clinical evidence to clarify how infection with Bbsl can trigger sustained immune dysregulation in a subset of patients. By integrating current data, we propose a conceptual model in which infection-induced immune activation may persist beyond microbial clearance, contributing to chronic inflammatory and autoimmune-like manifestations affecting multiple organ systems.

Viewing LD through this dual infectious–immunologic perspective has important diagnostic and therapeutic implications. Distinguishing active infection from post-infectious immune-mediated pathology remains essential to avoid both overtreatment and misdiagnosis. An integrated, mechanism-based approach may improve patient stratification and support the development of more targeted therapeutic strategies, ultimately contributing to more precise management of complex Lyme-associated conditions.

Future research should focus on identifying reliable biomarkers capable of distinguishing active infection from post-infectious immune-mediated pathology and on developing targeted immunomodulatory strategies for patients with persistent LD-associated manifestations. In addition, the potential contribution of coinfections with other tick-borne pathogens to persistent symptoms warrants further investigation, although current evidence remains limited and controversial.

## Figures and Tables

**Table 1:** Major Immunopathogenic Mechanisms in Post-Infectious Manifestations of LD.

Immunopathogenic Mechanism	Key Immune Components	Principal Biological Effects	Representative Disease Relevance
Dysregulated innate immune activation	TLR2 signaling, macrophages, dendritic cells	Excessive cytokine and chemokine production; amplification of inflammation	Acute and disseminated LD; priming of chronic inflammation
Type I IFN signaling	IFN-α/β, IFN-stimulated genes	Sustained inflammatory signaling independent of bacterial burden	Antibiotic-refractory LA; tissue-specific inflammation
Th1/Th17 polarization	CD4^+^ T cells, IFN-γ, IL-17	Persistent cellular inflammation; tissue injury	Chronic synovitis; neuroinflammatory phenotypes
Impaired immune regulation	Reduced regulatory T-cell activity	Failure of immune resolution following infection	Post-infectious inflammatory disease
Antigen persistence	Borrelial peptidoglycan	Chronic innate immune stimulation without viable organisms	Post-infectious LA
Infection-triggered autoimmunity	Molecular mimicry; epitope spreading	Expansion of autoreactive immune responses in susceptible hosts	Autoimmune-like post-infectious phenotypes

IFN-α/β—interferon alpha/beta; IFN-γ—interferon gamma; IL-17—interleukin-17; CD4—cluster of differentiation 4; LA—Lyme arthritis; TLR—Toll-like receptor; CD4—cluster of differentiation 4.

**Table 2:** System-Based Diagnostic Biomarkers and Supportive Markers in Lyme-Associated Clinical Manifestations.

Clinical Complication	Biochemical/Immunological Biomarker	Biological Sample	Immunological Relevance	Specificity
LNB	CXCL13	CSF	B-cell chemoattractant reflecting intrathecal B-cell recruitment and antibody production	Moderate (organ-specific)
	Intrathecal *Borrelia*-specific antibody index	CSF + serum	Demonstrates intrathecal synthesis of *Borrelia*-specific antibodies	High
	CXCL9	CSF	IFN-γ-induced chemokine reflecting Th1-mediated immune activation	Moderate
	CXCL10 (IP-10)	CSF	Marker of Th1-dominant chemokine signaling and T-cell recruitment	Moderate
	Neopterin	CSF or serum	Reflects macrophage activation and cellular immune response	Low–moderate
LA	*Borrelial* peptidoglycan	Synovial fluid	Persistent bacterial antigen driving innate immune activation and chronic synovial inflammation	Moderate (context-specific)
	IL-17	Synovial fluid or serum	Reflects Th17-mediated inflammatory pathways involved in chronic synovitis	Low–moderate
	RF	Serum	Autoantibody reflecting B-cell activation and autoimmune inflammatory responses	Low
	anti-CCP antibodies	Serum	Autoantibodies associated with citrullinated protein immune responses	High (for rheumatoid arthritis)
Lyme carditis	Cardiac troponin I/T	Serum	Marker of myocardial injury associated with inflammatory cardiac involvement	Low (nonspecific)
	NT-proBNP	Serum	Reflects myocardial stress and cardiac dysfunction	Low

LNB—Lyme neuroborreliosis; LA—Lyme arthritis; CSF—cerebrospinal fluid; CXCL—C-X-C motif chemokine ligand; IP-10—interferon-γ–inducible protein 10 (CXCL10); IL—interleukin; RF—rheumatoid factor; anti-CCP—anti–cyclic citrullinated peptide antibodies; NT-proBNP—N-terminal pro–B-type natriuretic peptide; ECG—electrocardiogram; AV—atrioventricular; CNS—central nervous system.

## Data Availability

No new data were created or analyzed in this study.
